# Acquisition of a large virulence plasmid (pINV) promoted temperature-dependent virulence and global dispersal of O96:H19 enteroinvasive *Escherichia coli*

**DOI:** 10.1128/mbio.00882-23

**Published:** 2023-05-31

**Authors:** Sydney L. Miles, Vincenzo Torraca, Zoe A. Dyson, Ana Teresa López-Jiménez, Ebenezer Foster-Nyarko, Damián Lobato-Márquez, Claire Jenkins, Kathryn E. Holt, Serge Mostowy

**Affiliations:** 1 Department of Infection Biology, London School of Hygiene and Tropical Medicine, London, United Kingdom; 2 Department of Infectious Diseases, Central Clinical School, Monash University, Melbourne, Victoria, Australia; 3 Wellcome Sanger Institute, Wellcome Genome Campus, Hinxton, United Kingdom; 4 Gastrointestinal Pathogens and Food Safety (One Health), UK Health Security Agency, London, United Kingdom; Institut Pasteur, Paris, France

**Keywords:** EIEC, zebrafish, host-pathogen interactions, evolution, *Shigella*, *Enterobacteriaceae*, virulence determinants

## Abstract

Enteroinvasive *Escherichia coli* (EIEC) and *Shigella* are closely related agents of bacillary dysentery. It is widely viewed that EIEC and *Shigella* species evolved from *E. coli* via independent acquisitions of a large virulence plasmid (pINV) encoding a type 3 secretion system (T3SS). Sequence Type (ST)99 O96:H19 *E. coli* is a novel clone of EIEC responsible for recent outbreaks in Europe and South America. Here, we use 92 whole genome sequences to reconstruct a dated phylogeny of ST99 *E. coli*, revealing distinct phylogenomic clusters of pINV-positive and -negative isolates. To study the impact of pINV acquisition on the virulence of this clone, we developed an EIEC-zebrafish infection model showing that virulence of ST99 EIEC is thermoregulated. Strikingly, zebrafish infection using a T3SS-deficient ST99 EIEC strain and the oldest available pINV-negative isolate reveals a separate, temperature-independent mechanism of virulence, indicating that ST99 non-EIEC strains were virulent before pINV acquisition. Taken together, these results suggest that an already pathogenic *E. coli* acquired pINV and that virulence of ST99 isolates became thermoregulated once pINV was acquired.

**IMPORTANCE:**

Enteroinvasive *Escherichia coli* (EIEC) and *Shigella* are etiological agents of bacillary dysentery. Sequence Type (ST)99 is a clone of EIEC hypothesized to cause human disease by the recent acquisition of pINV, a large plasmid encoding a type 3 secretion system (T3SS) that confers the ability to invade human cells. Using Bayesian analysis and zebrafish larvae infection, we show that the virulence of ST99 EIEC isolates is highly dependent on temperature, while T3SS-deficient isolates encode a separate temperature-independent mechanism of virulence. These results indicate that ST99 non-EIEC isolates may have been virulent before pINV acquisition and highlight an important role of pINV acquisition in the dispersal of ST99 EIEC in humans, allowing wider dissemination across Europe and South America.

## INTRODUCTION

Enteroinvasive *E. coli* (EIEC) and *Shigella* species are Gram-negative, human-adapted pathogens that cause bacillary dysentery. The greatest burden of bacillary dysentery is in low- and middle-income countries (LMICs) (1), although the true burden of EIEC infection is likely underestimated since it is difficult to distinguish from *Shigella*. Historically, *Shigella* was classified as its own genus, with four distinct species, but Multi-Locus Sequence Typing (MLST) and whole-genome sequencing data clearly show *Shigella* spp. are lineages of *E. coli*, as are EIEC (2, 3). Each *Shigella* and EIEC lineage evolved independently within the *E. coli* population, following the horizontal acquisition of a ~220 kbp virulence plasmid (also known as plasmid of invasion or pINV) from a currently unknown source (2). pINV encodes a type three secretion system (T3SS) that facilitates the invasion of human epithelial cells and is thermoregulated in both EIEC and *Shigella* (4).

A novel clone of EIEC, of serotype O96:H19 and Multi-Locus Sequence Type (ST) 99, was first described in 2012 in Italy and has since caused several foodborne outbreaks of moderate to severe diarrheal disease across Europe and South America (5 - 7). Before 2012, ST99 *E. coli* had not been reported in the literature as causing human disease but had been sporadically isolated from cattle and environmental sources (8). ST99 EIEC isolates have been characterized as possessing the virulence hallmarks of EIEC and *Shigella* (pINV and T3SS) (9), but its metabolic capacity closely resembles that of commensal *E. coli* and it has more recently been associated with *pga*-mediated biofilm formation (6, 9). It has therefore been proposed that ST99 EIEC diverged recently from ST99 *E. coli* due to the acquisition of pINV.

The zebrafish (*Danio rerio*) larvae model is widely used to study infection biology *in vivo* because of its rapid development and innate immune system that is highly homologous to that of humans (10, 11). Zebrafish have emerged as a valuable vertebrate model to study human enteropathogens like *Shigella* (12), highlighting the key roles of bacterial virulence factors (e.g., T3SS and O-antigen) (13, 14) and cell-autonomous immunity (e.g., autophagy and septin-mediated immunity) (12, 15) in host-pathogen interactions.

In this observation, we reconstruct a dated phylogeny of ST99 *E. coli* using publicly available whole genome sequences, to understand the role of pINV in its global dispersal. We develop a temperature-dependent zebrafish infection model to assess the virulence of EIEC and non-EIEC ST99 isolates, highlighting the power of zebrafish infection in studying the evolution of novel enteropathogens causing disease in humans.

### ST99 EIEC diverged ~40 years ago

**Fig 1:**
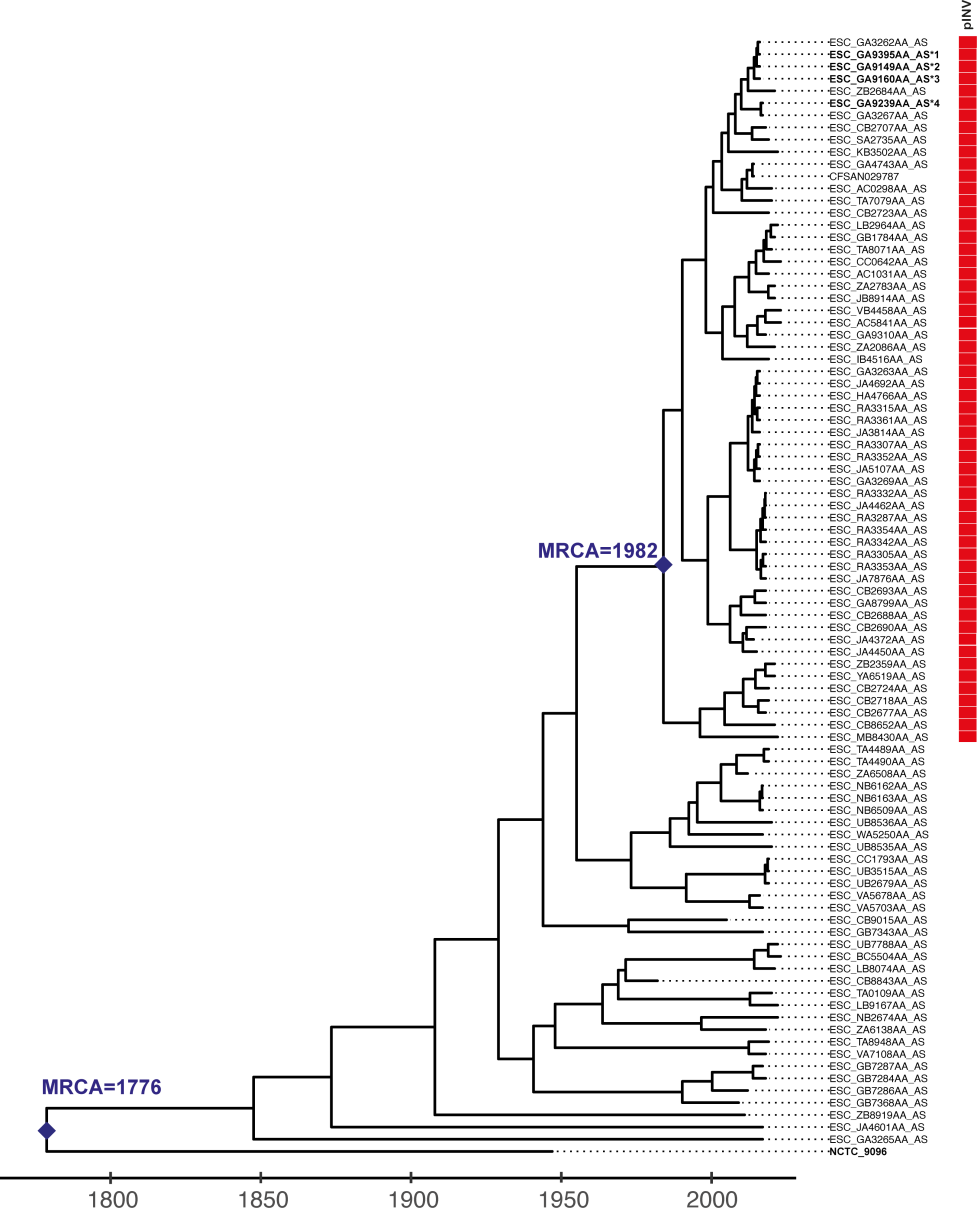
Time-calibrated phylogeny of 92 Sequence Type (ST)99 genomes. BactDating was used to infer a time-calibrated phylogeny, incorporating the output from the recombination detection software, Gubbins. Blue diamonds indicate the internal nodes representing the most recent common ancestors (MRCA) of interest. Tip labels represent assembly barcodes correlating to the isolate accession in Enterobase. Tip labels in bold represent isolates that we tested *in vivo*. As determined using ShigEiFinder (20), the presence of the invasion plasmid (pINV) is indicated by a red box in the pINV column. We estimate the MRCA of the whole group to be ~1776 and the MRCA for the pINV+ cluster to be ~1982.

To dissect the evolution of the ST99 clone and its transition to EIEC, we analyzed all publicly available ST99 genomes (*n* = 92), using the EnteroBase integrated software environment (16). EnteroBase routinely scans short-read archives and retrieves *E. coli* and *Shigella* sequences from the public domain or uses user-uploaded short reads. We used Gubbins v.3.2.1 (17) to filter recombinant sites, RaxML v.8.10 to infer a Maximum Likelihood phylogenetic tree and BactDating v.1.2 (18) to date the phylogeny (Fig. 1), as previously described by Didelot and Parkhill (19). Root-to-tip genetic distances were positively associated with the year of isolation (*R^2^* = 0.19, *P* = 6 × 10^-3^), and the date-randomization test showed no overlap between results of observed and date-randomized analyses (Fig. S1), indicating a moderate molecular clock signal to support dating analysis. From this analysis, we estimate that the most recent common ancestor (MRCA) of the whole ST99 group (pINV+ and pINV–) existed circa 1776 [95% highest posterior density (HPD), 1360–1927]. To test for the presence of pINV, we used ShigEiFinder, which scans the genomes for pINV-encoded genes and deems an isolate positive for pINV when 26 of 38 genes are present (20). The pINV+ isolates form a distinct cluster, with their MRCA existing circa 1982 (95% HPD, 1965–2011) (Fig. 1). This suggests that the ST99 EIEC may have been circulating undetected for ~30 years before being detected in the 2012 outbreak.

To test the role of pINV in the dispersal of ST99 EIEC, we selected: (i) four recent pINV+ isolates from moderate-to-severe diarrheal outbreaks in the United Kingdom in 2014 and 2015 (21, 22) to represent contemporary ST99 EIEC, (ii) a Congo red-negative colony to represent a T3SS-deficient strain isogenic strain, and (iii) the oldest available ST99 isolate (~1945, NCTC 9096, pINV−) to represent ancestral pINV− ST99 (see Fig. 1).

### ST99 EIEC virulence is temperature-dependent in zebrafish

**Fig 2:**
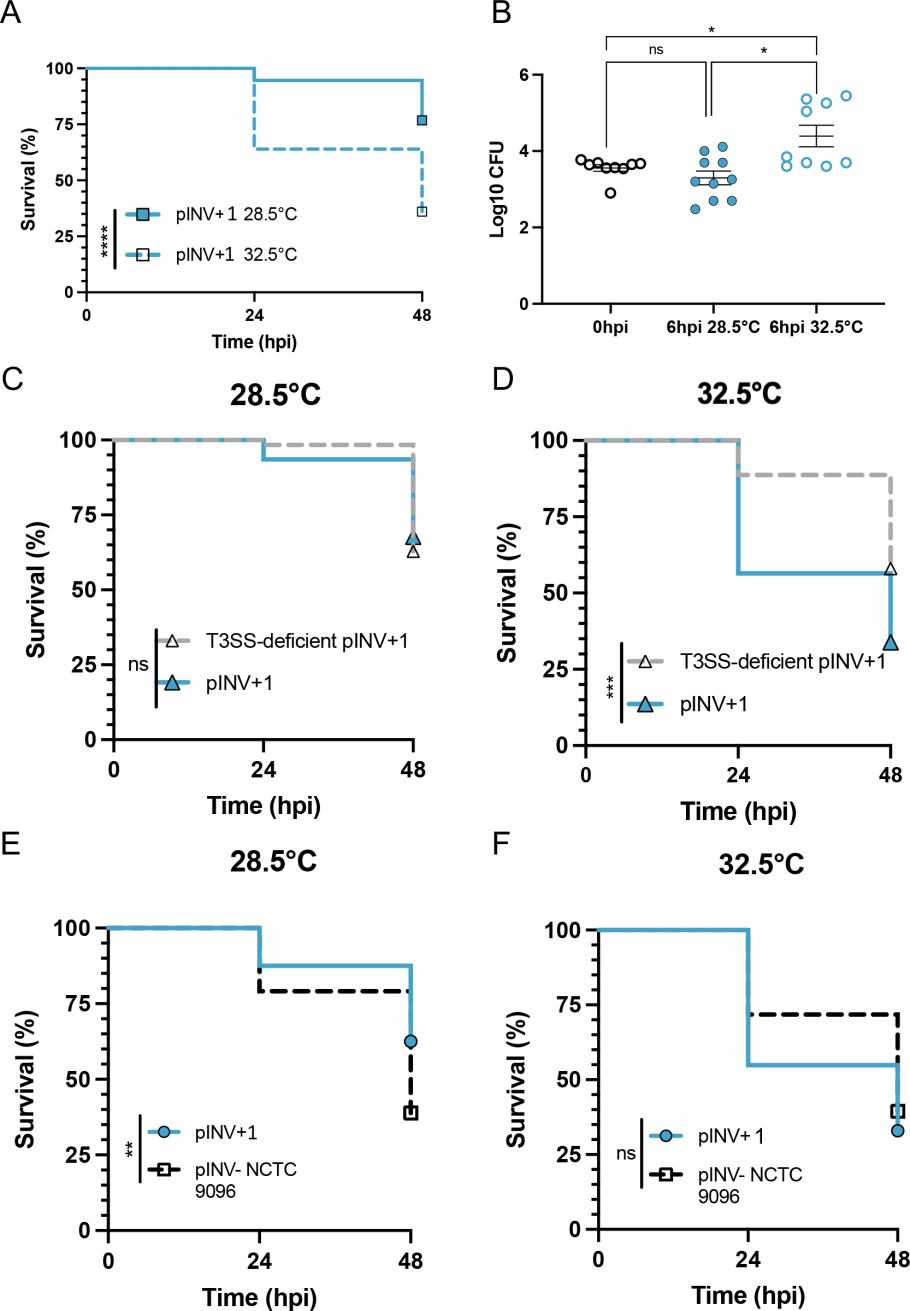
Temperature-dependent and -independent mechanisms of virulence in the ST99 group. Zebrafish larvae at 3 d post-fertilization were injected with 5,000 CFU of a representative pINV+ ST99 strain, a T3SS-deficient strain and an ancestral pINV− ST99 strain, before being separated for incubation at 28.5°C or 32.5°C. (**A, B**) pINV+1 strain exhibits a temperature-dependent virulence with significantly more killing observed at 32.5°C. Enumeration of bacterial burden is also temperature dependent, with greater CFUs quantified at 6 h post-infection from larvae incubated at 32.5°C. Black circles indicate pINV+1 CFUs at 0 hpi, blue filled circles indicate pINV+1 CFUs at 6 hpi incubated at 28.5°C, and blue outlined circles indicate pINV+1 CFUs at 6 hpi incubated at 32.5°C. (**C, D**) Thermoregulated virulence is lost in a T3SS-deficient (Congo red negative) pINV+1 strain (gray dashed line). (**E, F**) pINV− strain NCTC 9096 (black dashed line) is virulent in the zebrafish model in a non-temperature-dependent manner. Significance was tested using Log-rank (Mantel-Cox) test for survival curves. For CFUs (panel B), significance was tested using a one-way ANOVA with Sidak’s correction. *P < 0.0332; **P < 0.0021; ***P < 0.0002; and ****P < 0.0001.

The zebrafish infection model has generated fundamental advances in our understanding of *Shigella* and its ability to infect humans (23). To test the virulence of pINV+ ST99 EIEC strains, ~5,000 CFU was injected into the hindbrain ventricle (HBV) of zebrafish larvae at 3 d post-fertilization (dpf) (Fig. S1A). Infected zebrafish larvae are typically incubated at 28.5°C for optimal development but we have shown they can also be maintained at 32.5°C (13), allowing the study of temperature-dependent virulence. For the pINV+ strains, we observed ~75% survival when larvae were incubated at 28.5°C but only ~30% survival when incubated at 32.5°C (Fig. 2A; Fig. S2B and C). In agreement with survival results, CFUs recovered at 6 h post-infection (hpi) were significantly lower at 28.5°C than CFUs recovered at 32.5°C (Fig. 2B; Fig. S2D and E), suggesting that larvae were more able to control infection at 28.5°C.

To test if the T3SS in ST99 EIEC is functional and thermoregulated, we compared the secretion of virulence factors by ST99 EIEC and *Shigella flexneri in vitro* (Fig. S3). The overall abundance of secreted proteins is lower for ST99 EIEC as compared to *S. flexneri*, but the relative abundance of major secreted effectors appears similar. One exception is SepA, a protein secreted independently of the T3SS, whose presence is known to be variable in other EIEC lineages (24). Although we do not observe significant differences in secretion between 28.5°C and 32.5°C under these *in vitro* conditions tested, the T3SS in ST99 EIEC is clearly thermoregulated (with optimal secretion *in vitro* at 37°C).

Having established a temperature-dependent EIEC-zebrafish infection model, it was next of great interest to test the virulence of an isogenic, T3SS-deficient ST99 EIEC strain and an ancestral pINV− ST99 isolate. Since we observed no significant differences in zebrafish survival or bacterial burden between the four pINV+ strains at either 28.5°C or 32.5°C (Fig. S2B to E), we chose one isolate (pINV+1) as a representative pINV+ isolate to compare with the T3SS-deficient and pINV− isolate (NCTC 9096).

### ST99 *E. coli* comprises temperature-dependent and -independent mechanisms of virulence

To test if the virulence of ST99 *E. coli* in zebrafish is dependent on the acquisition of pINV and the T3SS, we selected a naturally T3SS-deficient colony (Congo red negative) to compare against pINV+1. Colonies were screened for several pINV-encoded genes by colony PCR and found to be deficient in genes located in the T3SS-encoding region of pINV (*mxiG*, *mxiD,* and *icsB*), but positive for genes located outside (*ospF* and *ipaH*) (Fig. S4). In addition, we verified dysfunction of the T3SS, showing that T3SS effector proteins are not secreted by T3SS-deficient colonies at 37°C but are by the wild-type EIEC isolate (Fig. S3). These results suggest that the T3SS-encoding region has been lost in Congo red negative colonies, consistent with what has previously been reported for *S. flexneri* (25). Infection of zebrafish with these isolates shows that the thermoregulated virulence is lost in the T3SS-deficient strain, with no significant difference in zebrafish survival observed between 28.5°C and 32.5°C, whilst thermoregulated virulence is maintained in the wild-type strain (Fig. 2C and D; Fig. S5A and B). These result implicate acquisition of the T3SS (and pINV) in the temperature-dependent virulence of pINV+1.

Next, we compared the virulence of a non-EIEC (pINV−) ST99 isolate (NCTC 9096) and a pINV+ EIEC isolate (pINV+1) strains using our EIEC-zebrafish infection model. Strikingly, NCTC 9096 was significantly more virulent than pINV+1 at 28.5°C, with only ~35% of infected larvae surviving at 48 hpi (Fig. 2E). Although no change in survival of larvae infected with NCTC 9096 is observed at 32.5°C (as compared to that of 28.5°C), survival of pINV+1 infected larvae significantly decrease at 32.5°C, consistent with a role for temperature-dependent virulence. At 32.5°C, we found that both pINV+1 and NCTC 9096 isolates were equally virulent (Fig. 2F).

The trend in virulence was also reflected in the quantification of bacterial burden (Fig. S5C and D). When incubated at 32.5°C, we observed a ~2 log increase in pINV+1 CFUs enumerated from larvae at 6 hpi but not when incubated at 28.5°C. We observe a similar increase in NCTC 9096 CFUs quantified, irrespective of temperature. These results show temperature-dependent virulence of the pINV+1 strain and non-temperature-dependent virulence of the pINV− strain, consistent with our observations for the T3SS-deficient EIEC isolate.

## DISCUSSION

It is widely recognized that the acquisition of pINV is a defining feature in the evolution of EIEC and *Shigella* (26). Here, we analyze the evolution of ST99 EIEC and propose that an MRCA for the pINV+ group existed in the early 1980s. This suggests that ST99 EIEC may have been circulating undetected for ~30 years until it was implicated in the 2012 outbreak in Italy, perhaps because EIEC infections are typically endemic in regions where surveillance and sequencing of enteropathogens are limited.

We prove that the virulence of ST99 EIEC strains is thermoregulated *in vitro* and *in vivo* (with zebrafish larvae less able to control infection), leading to increased killing and greater bacterial replication at 32.5°C. Some killing is still observed at 28.5°C, suggesting a low-level activation of the T3SS and/or non-T3SS mechanisms of virulence *in vivo*, which would be of interest to test in future studies. These data are consistent with previous reports for pINV-mediated virulence in both *S. flexneri* and *Shigella sonnei* (4, 13). Our zebrafish infection model highlights the importance of temperature in EIEC virulence and supports the hypothesis that pINV acquisition is the first key step in the evolutionary pathway toward becoming a human-adapted pathogen. Our data using zebrafish infection further show that non-EIEC ST99 isolates can also cause disease and that the ability of the ST99 clone to cause disease does not strictly rely on the acquisition of pINV and the transition to EIEC. Considering that pINV− ST99 strain NCTC 9096 is highly virulent *in vivo*, we conclude that it must encode separate, non-thermoregulated mechanism(s) of virulence that becomes less important for human infection once pINV is acquired.

Collectively, our findings illuminate the short history of ST99 EIEC and implicate pINV acquisition as a key factor in its epidemiological success. Our approach also reveals a separate, non-thermoregulated virulence mechanism in a pINV− ST99 isolate, suggesting that an already pathogenic *E. coli* may have acquired pINV. Further studies, including identifying the source of pINV and those isolates likely to acquire it, are important to fully understand and prevent the dispersal of novel EIEC and *Shigella* clones infecting humans.

## METHODS

### Bacterial strains

Four pINV+ EIEC strains isolated in diarrhoeal outbreaks from the United Kingdom in 2014/2015 were included in this study (Table 1), and strains were identified and sequenced through routine surveillance and kindly shared with us by the UK Health and Security Agency (UKHSA). A pINV− ST99 strain (Table 1) included in this study was obtained from the National Culture Type Collection (NCTC). *S. flexneri* M90T was used as a positive control for the *in vitro* secretion assay (27).

**TABLE 1:** Bacterial strains used in experimental work/as a reference genome^*a*^

Strain	Source	Serotype	pINV	Origin	Sequence accession no.	Enterobase assembly barcode
NCTC 9096	NCTC	O96:H19	−	Denmark, 1945	UGEL01000000	ESC_CC4859AA_AS
pINV+1	UKHSA	O96:H19	+	United Kingdom (Travel to Turkey), 2014	SRR3578973	ESC_GA9395AA_AS
pINV+2	UKHSA	O96:H19	+	Kingdom (Travel to Turkey), 2014	SRR3578582	ESC_GA9149AA_AS
pINV+3	UKHSA	O96:H19	+	Kingdom (Travel to Turkey), 2014	SRR3578593	ESC_GA9160AA_AS
pINV+4	UKHSA	O96:H19	+	United Kingdom, 2015	SRR3578770	ESC_GA9239AA_AS
CFSAN029787	NA	O96:H19	+	Italy, 2012	Chromosome: CP011416.1, pINV: CP011417.1	ESC_GA4743AA_AS
*S. flexneri* M90T	Institut Pasteur	5a	+	Mexico, 1955	NA^*b*^	NA^*b*^

^*a*^ Strains NCTC 9096 and the four pINV+ ST99 strains obtained from the UKHSA were used for the *in vivo* work. CFSAN029787 was used as a reference strain for the phylogenomic analyses. Enterobase assembly accessions correlate to tip labels in the dated phylogeny (Fig. 1).

^*b*^ NA, not applicable.

To obtain a T3SS-deficient ST99 isolate, bacteria (pINV+1) were grown on trypticase soy agar (TSA) plates supplemented with 0.01% Congo red (Sigma-Aldrich) dye. A white colony was selected as a natural isogenic mutant, unable to secrete T3SS effector proteins, as previously described (28). We tested this isolate for the presence of five pINV-encoded genes (*mxiG*, *mxiD, icsB, ipaH,* and *ospF*) by colony PCR, using primers described in Table 2.

**TABLE 2:** Primers used to detect for the presence of pINV-encoded genes

Primer name	Primer sequence (5'-3')
mxiD_Fwd	CAGAATGTAAGTAATGCACTGGCTATGATAC
mxiD_Rev	CTGTCTATAAAATCCTGATCTAGAGGAAGGTTATC
mxiG_Fwd	CTGATTGTTGGGATAAGGCTGG
mxiG_Rev	CCGAGATCCCCTGTTTACCTC
ospF_Fwd	AAAAGATGAAGGCCTGATGGGAGCATTAAC
ospF_Rev	TGGTGGATAAAACCCGCCAGAATGAACA
icsB_Fwd	GGTTCCAAGATCTGGCGATTTAAGAGAATTGTAATAATC
icsB_Rev	GGGCCTATACGCGTTGAAGATACAGAG
ipaH1.4_Fwd	GGGCATGAAAAAAGCTACATCC
ipaH1.4_Rev	CACCATTATTCGAGTATAGGGAGAG

### Genomic analysis

Enterobase was used to identify publicly available ST99 genomes, using the filter by ST function (16); all ST99 genomes with an associated assembly and isolation date were included in our study, sequence accessions and metadata can be found in Table S1. Complete genome sequences of strains NCTC 9096 and CFSAN029787 were downloaded from GenBank (accessions UGEL01000000 and CP011416.1, respectively). All genomic analyses were performed using the Cloud Infrastructure for Microbial Bioinformatics (CLIMB) (29). Snippy v.4.6 (https://github.com/tseemann/snippy) was used to generate a core genome alignment, using CFSAN029787 as the reference. Gubbins v.3.2.1 (17) was used to identify recombinant regions of the alignment, and RaxML v.8.10 (30) was used to build a maximum likelihood phylogenetic tree, using the General Time Reversible (GTR) GAMMA nucleotide substitution model. BactDating v.1.2 (31) was used to infer the dated phylogeny, using the “relaxedgamma” model, the option to incorporate Gubbins detected recombination was selected and 10^5^ Markov chain Monte Carlo (MCMC) chain iterations were run. To confirm the temporal signal (association between genetic divergence and time) within our dataset, tip nodes were assigned random dates and the analysis was rerun (this was completed *n* = 10 times). We saw no overlap between the substitution rates of our real data and the randomized datasets (Fig. S3) that shows that the data pass the stringent test CR2 for the presence of a temporal signal according to Duchene et al. (32). To screen assemblies for the presence of pINV, ShigEiFinder was used, which screens for 38 pINV-encoded genes and deems an isolate positive when at least 26 genes are present (20).

### Inoculate preparation

Single red colonies (pINV+ EIEC) or white colonies (pINV− NCTC 9096 and T3SS-deficient EIEC) were selected and inoculated into 5 mL trypticase soy broth (TSB) and incubated overnight at 37°C, shaking at 400 rpm. 400 µL of overnight culture was subsequently diluted in 20 mL TSB and grown until an optical density of ~0.6 (measured at 600 nm) was reached. For zebrafish larvae infections, inoculate preparation was carried out by resuspension of the bacteria at the desired concentration in phosphate buffer saline (PBS, Sigma-Aldrich) pH 7.4 containing 2% polyvinylpyrrolidone (Sigma-Aldrich) and 0.5% phenol red (Sigma-Aldrich) as previously described (13).

### Zebrafish larvae infection

Wild-type-AB zebrafish embryos were used for *in vivo* studies. Embryos were kept in 0.5 × E2 medium supplemented with 0.3 µg/mL methylene blue and incubated at 28.5°C unless otherwise stated. Using a microinjector, ~1 nL of bacterial suspension was injected into the HBV of 3 d post-fertilization (dpf) zebrafish larvae, following previously described procedures (13). The precise inoculum was determined retrospectively by homogenization of larvae at 0 h post-infection and plating on TSA plates supplemented with 0.01% Congo red.

For survival assays, zebrafish larvae were visualized using a light stereomicroscope at 24 and 48 hpi; the presence of a heartbeat was used to determine viability. For colony forming unit (CFU) counts, larvae were disrupted in PBS using a pestle pellet blender at 0 and 6 hpi. Serial dilutions in PBS and plating on TSA plates supplemented with 0.01% Congo red were then performed to estimate the bacterial load in each larva. Statistical analysis was performed in GraphPad Prism 9.

### *In vitro* secretion assay

Secretion of T3SS effectors was tested as previously described (33). Briefly, bacteria were grown overnight, subcultured and grown until exponential phase (OD = 0.4–0.5) at either 28.5°C, 32.5°C, or 37°C. Cultures were then incubated for 3 h in the presence or absence of Congo red to induce type 3 secretion. Secreted proteins were collected from culture supernatants, precipitated using trichloroacetic acid (Sigma-Aldrich), and then analyzed using SDS-PAGE and Coomassie Brilliant Blue R-250 (Bio-Rad) staining.

## Data Availability

All genomes used in this study are publicly available in Enterobase, and assembly accessions are provided in Table S1.

## SUPPLEMENTAL MATERIAL

Fig S1 mbio.00882-23-s0001.pdf Results of date randomization test.

Fig S2 mbio.00882-23-s0002.pdf Virulence of ST99 EIEC isolates is temperature dependent.

Fig S3 mbio.00882-23-s0003.pdf Secretion of virulence factors *in vitro* by ST99 EIEC pINV+1 (Congo red+ colony), T3SS-deficient ST99 EIEC (Congo red- colony), and *S. flexneri.*

Fig S4 mbio.00882-23-s0004.pdf Colony PCR to check for the presence of pINV-encoded genes.

Fig S5 mbio.00882-23-s0005.pdf Bacterial burden of pINV+1 compared to its T3SS-deficient counterpart and the oldest available pINV− isolate, NCTC 9096.

TABLE S1 mbio.00882-23-s0006.xlsx Details and metadata for all genomes downloaded from Enterobase.

ASM does not own the copyrights to Supplemental Material that may be linked to, or accessed through, an article. The authors have granted ASM a non-exclusive, world-wide license to publish the Supplemental Material files. Please contact the corresponding author directly for reuse.
