# Supplementary material for: Acquisition of a large virulence plasmid (pINV) promoted temperature-dependent virulence and global dispersal of O96:H19 enteroinvasive Escherichia coli
Source: mBio. 2023 May 31;14(4):e00882-23. doi: 10.1128/mbio.00882-23 (PMC10470518; doi:10.1128/mbio.00882-23)
Supplement: Fig S1 — Results of date randomization test. [file mbio.00882-23-s0001.pdf]

Figure S1. Results of date randomisation test.

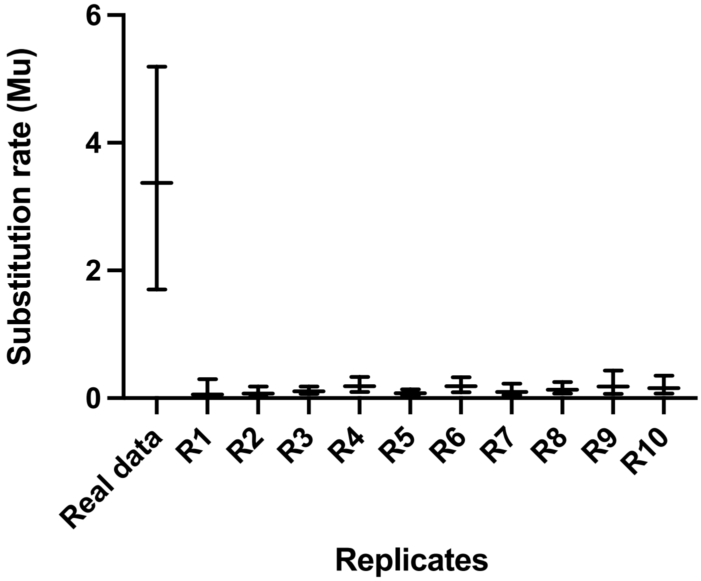

Figure S1. Results of date randomisation test. To test for temporal signal within our dataset, isolation dates were randomised n=10 times and the analysis was re-run (R1-R10). We saw no overlap with the substitution rate of our real data, indicating the suitability of our dataset for performing a dated phylogenomic analysis.
