# Supplementary material for: Acquisition of a large virulence plasmid (pINV) promoted temperature-dependent virulence and global dispersal of O96:H19 enteroinvasive Escherichia coli
Source: mBio. 2023 May 31;14(4):e00882-23. doi: 10.1128/mbio.00882-23 (PMC10470518; doi:10.1128/mbio.00882-23)
Supplement: Fig S2 — Virulence of ST99 EIEC isolates is temperature dependent. [file mbio.00882-23-s0002.pdf]

Figure S2. Virulence of ST99 EIEC isolates is temperature dependent.

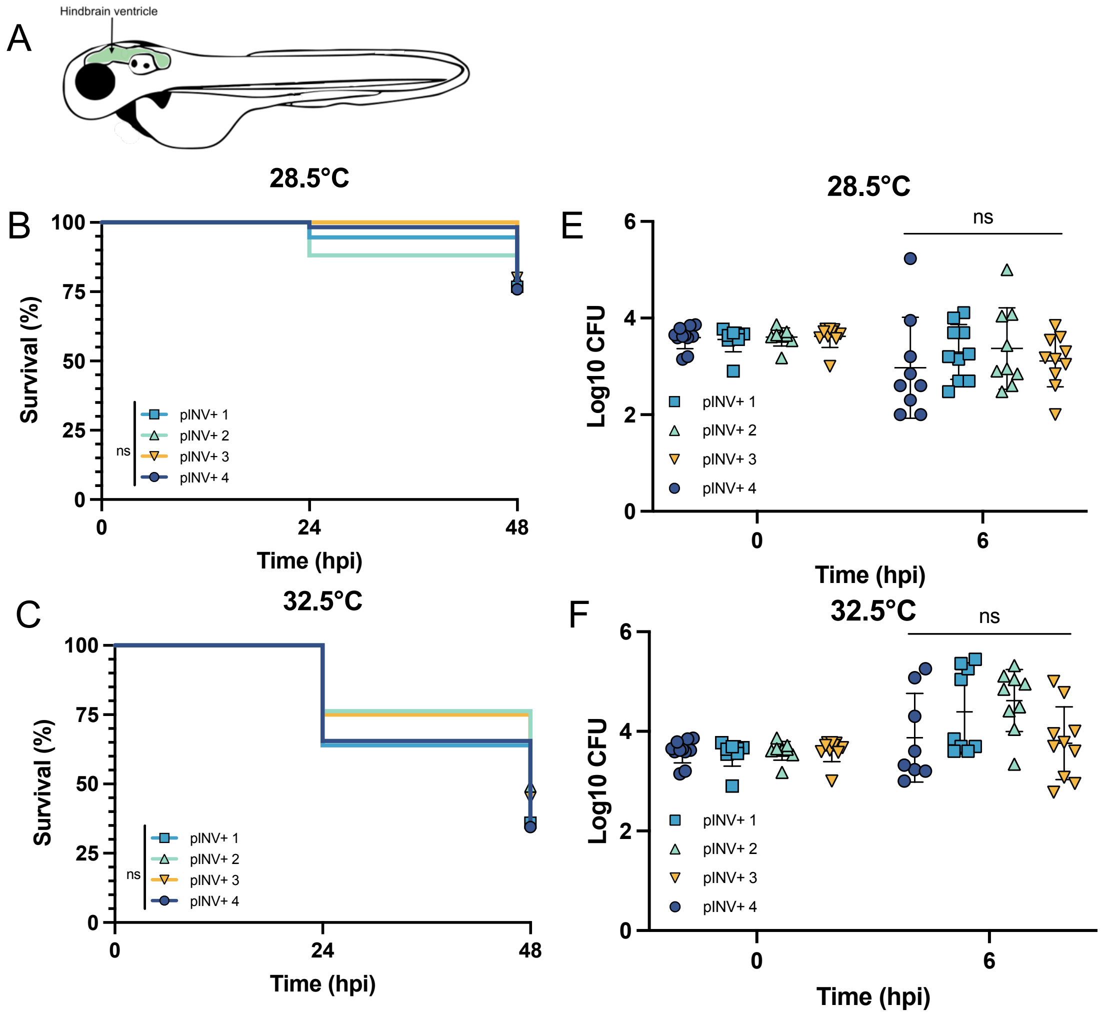

Figure S2. Virulence of ST99 EIEC isolates is temperature dependent. A) Schematic of a zebrafish larva, indicating the hindbrain ventricle as the infection site. **B-E)** 3-day post fertilisation larvae were injected with 5000 CFU of bacteria before being separated for incubation at 28.5°C or 32.5°C. **B,C)** pINV+ ST99 strains exhibit a temperature-dependent virulence, with significantly more killing observed at 32.5°C. No differences in survival were observed between strains (p= 0.22). Significance was tested using Log-rank (Mantel-Cox) test. **D,E**). Enumeration of bacterial burden is also temperature dependent in pINV+ strains, with greater CFUs quantified at 6 hours post infection from larvae incubated at 32.5°C. Significance for CFUs was tested using a two-way ANOVA with Sidak's correction.
