# Supplementary material for: Acquisition of a large virulence plasmid (pINV) promoted temperature-dependent virulence and global dispersal of O96:H19 enteroinvasive Escherichia coli
Source: mBio. 2023 May 31;14(4):e00882-23. doi: 10.1128/mbio.00882-23 (PMC10470518; doi:10.1128/mbio.00882-23)
Supplement: Fig S3 — Secretion of virulence factors in vitro by ST99 EIEC pINV+1 (Congo red+ colony), T3SS-deficient ST99 EIEC (Congo red- colony), and S. flexneri. [file mbio.00882-23-s0003.pdf]

**Figure S3. *S*ecretion of virulence factors *in vitro* by ST99 EIEC pINV+1 (Congo red+ colony), T3SS-deficient ST99 EIEC (Congo red- colony) and *S.flexneri*.**

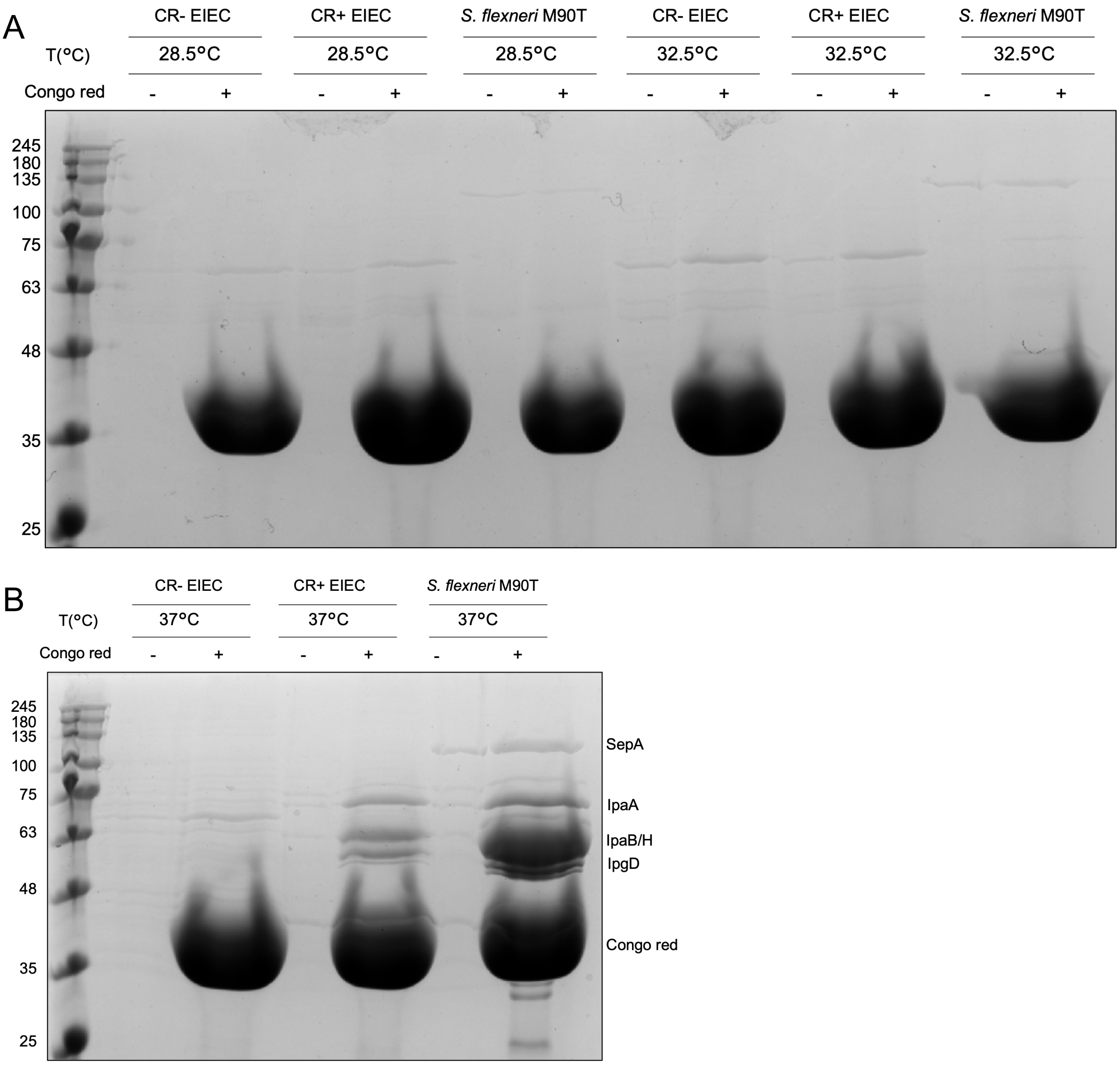


**Figure S3. *S*ecretion of virulence factors *in vitro* by ST99 EIEC pINV+1 (Congo red+ colony), T3SS-deficient ST99 EIEC (Congo red- colony) and *S.flexneri*.** SDS-PAGE gel stained with Coomassie blue, showing secreted factors in the presence or absence of Congo red, at different temperatures (A: 28.5°C and 32.5°C, B: 37°C). *S. flexneri* M90T is used as a positive control. Several well characterised secreted virulence factors are identified and labelled based on their abundance and molecular weight.
