# Supplementary material for: Acquisition of a large virulence plasmid (pINV) promoted temperature-dependent virulence and global dispersal of O96:H19 enteroinvasive Escherichia coli
Source: mBio. 2023 May 31;14(4):e00882-23. doi: 10.1128/mbio.00882-23 (PMC10470518; doi:10.1128/mbio.00882-23)
Supplement: Fig S4 — Colony PCR to check for the presence of pINV-encoded genes. [file mbio.00882-23-s0004.pdf]

Figure S4. Colony PCR to check for the presence of pINV encoded genes.

A

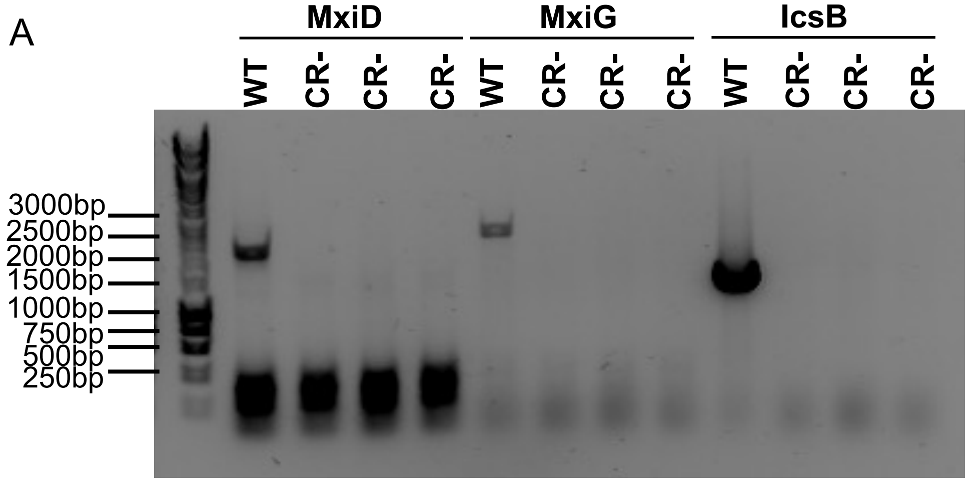

B

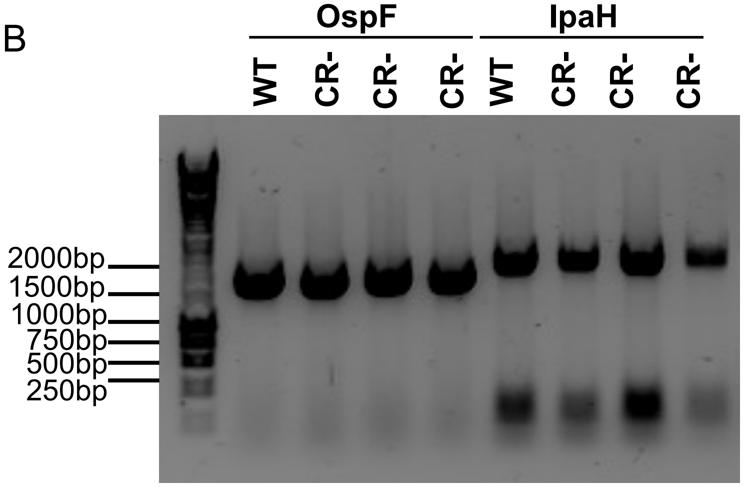

Figure S4. Colony PCR to check for the presence of pINV encoded genes. We performed colony PCR to check Congo red negative (CR-) pINV+1 colonies for (A) the presence of genes located in the T3SS-encoding region (*mxiD, mxiG* and *icsB*) and (B) outside of the T3SS-encoding region (*ospF* and *ipaH*). We see the loss of genes located in the T3SS-encoding region in CR- colonies, but not the wild type, indicating that this region has been lost.
