# Supplementary material for: Acquisition of a large virulence plasmid (pINV) promoted temperature-dependent virulence and global dispersal of O96:H19 enteroinvasive Escherichia coli
Source: mBio. 2023 May 31;14(4):e00882-23. doi: 10.1128/mbio.00882-23 (PMC10470518; doi:10.1128/mbio.00882-23)
Supplement: Fig S5 — Bacterial burden of pINV+1 compared to its T3SS-deficient counterpart and the oldest available pINV− isolate, NCTC 9096. [file mbio.00882-23-s0005.pdf]

**Figure S5. Bacterial burden of pINV+1 compared to its T3SS-deficient counterpart and the oldest available pINV- isolate, NCTC 9096.**

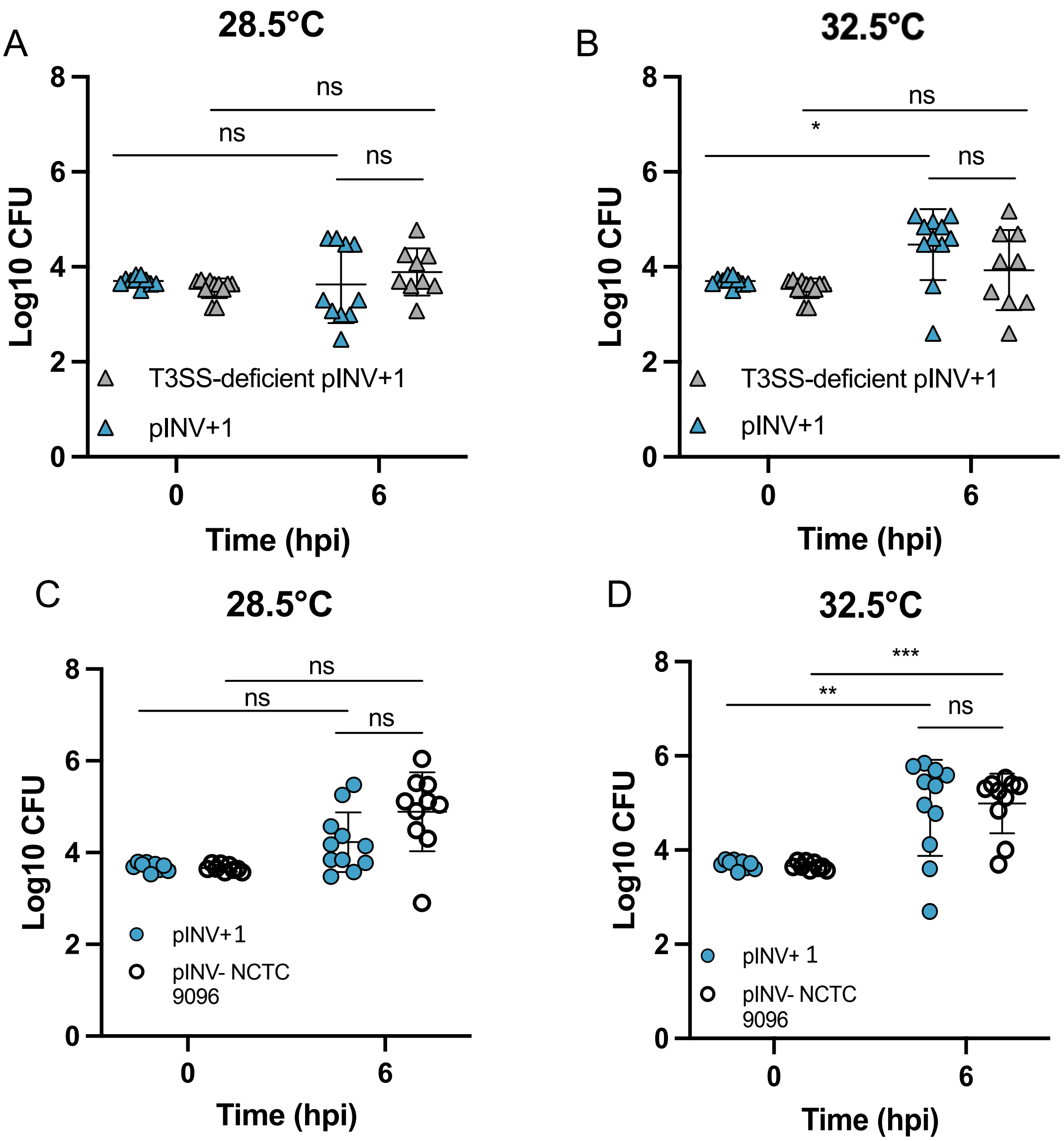


**Figure S5. Bacterial burden of pINV+1 compared to its T3SS-deficient counterpart and the oldest available pINV- isolate, NCTC 9096. A,B)** We observe a temperature dependent increase in CFUs enumerated from larvae infected with pINV+1 at 32.5°C, but not for its T3SS-deficient counterpart. **C,D)** Enumeration of CFUs from infected larvae at 6 hpi is temperature dependent in the case of pINV+1, but not pINV- NCTC 9096. Significance is tested using a one-way ANOVA with Sidak's correction. *$p<0.0332$;**$p< 0.0021$;***$p<0.0002$;****$p<0.0001$.
